# Scaling postpartum family planning services in the Democratic Republic of Congo: outcomes and lessons learned

**DOI:** 10.1136/bmjsrh-2023-202114

**Published:** 2024-01-30

**Authors:** Rita Kabra, Komal Preet Allagh, Brigitte Nsiku Kini, Robert Mulunda Kanke, James Kiarie

**Affiliations:** 1 Department of Sexual and Reproductive Health and Research, including UNDP/ UNFPA/UNICEF/WHO/ World Bank Special programme of Research, Development and Research Training in Human Reproduction, World Health Organization, Geneva, Switzerland; 2 Consultant, Department of Sexual and Reproductive Health and Research, World Health Organization, Geneva, Switzerland; 3 World Health Organization, Kinshasa Gombe, Democratic Republic of Congo

**Keywords:** Counseling, long-acting reversible contraception, family planning services

## Why was change needed?

Maternal, neonatal and infant mortality rates in the Democratic Republic of Congo (DRC) are among the highest in the world. Although the maternal mortality rate has decreased since 2000, there were still a total of 16 000 maternal deaths reported in 2017 from the DRC.^1^ In 2020, the maternal mortality rate in DRC increased to 547 deaths per 100 000 live births, a 16% increase from 2017.^2^ Additionally, the neonatal, infant and child mortality rates are also high, at 28, 58 and 104 deaths per 1000 live births, respectively.^1^ Family planning (FP) plays a key role in reducing these high mortality rates, especially if it is initiated early, such as immediately after childbirth. The Multiple Indicator Cluster Survey (MICS) 2016–2018 shows that only 17.6% of women of reproductive age in the DRC use modern contraceptives, while 28.7% have an unmet need for FP.^3^ Among currently married women, there is a 57% demand for FP, especially postpartum, when 37% of women wish to delay and space births, while 20% want to limit births.^3^ The overall postpartum family planning (PPFP) uptake in the DRC, at 6 months following childbirth, is only 18%.^4^ Several studies have shown that strengthening PPFP during antenatal care, postnatal care, childbirth and immunisation services can improve maternal and child health, increase contraceptive prevalence, and effectively address the unmet need for FP.^5 6^

## How did we go about implementing change?

The Yam Daboo study trial was conducted in Burkina Faso and DRC (Kinshasa province) from 2015 to 2018 to strengthen PPFP services at primary healthcare facilities and determine the effectiveness of the low-cost PPFP intervention package in increasing contraceptive uptake in the first year postpartum.^7^ At 12 months’ postpartum, 46% of the intervention group women in DRC used modern contraceptives, compared with 35% of the control group. The intervention group had a four times higher uptake of long-acting reversible contraceptives (LARCs), with 22% using implants as compared with 6% in the control group.^7^ The success of the study was attributed to the integration of counselling for PPFP in the midwifery curriculum, resulting in counselling for PPFP and increased contraceptive uptake.

After consultation with heads of the provincial health divisions, heads of the midwifery section of the Instituts Supérieurs des Techniques Médicales (ISTM), and supporting partners from Soins de Santé Primaires en Milieu Rural (SANRU), the National Reproductive Health Programme (Programme National de Santé de la Reproduction, PNSR) decided to scale up the Yam Daboo study interventions in the Kasaï Region of the DRC, specifically in the provinces of Kasaï, Kasaï Central and Kasaï Oriental, with support from the WHO, under the FP Accelerator Project.^8^ The 2018 MICS survey shows that there is a significant need for FP in these provinces as they have low uptake of modern contraceptives, high unmet need for FP, and high demand for spacing between births, as shown in table 1.

**Table 1:** Comparison of modern contraceptive uptake, unmet need and demand for spacing births between provinces

Province	mCPR (%)	Unmet need for FP (%)	Demand for spacing births (%)
Kasaï	13.9	48.5	25.9
Kasaï Oriental	3.8	21.8	28.2
Kasaï Central	8.4	22.4	38.0

FP, family planning; mCPR, modern contraceptive prevalence rate.

We implemented the following interventions to scale up PPFP services in the Kasaï region: (i) updated and adapted the PPFP counselling tool^9^ to the local context (January 2020), (ii) training of trainers on the use of the PPFP counselling tool (March 2020), (iii) training of midwifery teachers from the three ISTMs on the PPFP counselling tool (March 2020), (iv) developed pedagogical notes on FP counselling and pilot-tested these on final-year midwifery students (March 2020) and (v) incorporated the PPFP counselling tool into the undergraduate midwifery training programme (in Kasaï, Kasaï Oriental in 2020 and in Kasaï Central in 2021).

In January 2020, the PPFP counselling tool was updated and adapted to the local context. With support from WHO, the National Reproductive Health Programme (PNSR) organised training of trainers’ sessions on the PPFP counselling tool for provincial reproductive health coordinators, experts in the provincial health division, and the heads of the midwifery section from the ISTMs in March 2020. To ensure that the approach is sustainable, the adapted PPFP counselling tool was integrated into the basic curriculum of the midwives' training programme at ISTM in the three provinces. To achieve this, 20 ISTM midwifery teachers from the three provinces were first trained on PPFP (benefits of pregnancy spacing and planning for health, latest WHO recommendations for contraceptive options in PPFP/post-abortion family planning (PAFP) and the use of the PPFP counselling tool. All participants were engaged in role-playing exercises and could effectively use the tool by the end of the training sessions. Additional training was given on using the Medical Eligibility Criteria for Contraceptive Use (MEC) Wheel and on integrating FP into their teaching curriculum. The PPFP methods available in DRC include copper intrauterine device (IUD), implants, oral contraceptive pills, injectables and tubal ligation. The trained teachers from each province developed pedagogical notes for the chapter on FP counselling, emphasising the significance of counselling and the use of the PPFP counselling tool and MEC Wheel. Interestingly, the pedagogical notes developed by the three teams were almost identical. The notes were pilot-tested on 53 final-year midwifery students across the three provinces (20 in Kasaï, 21 in Kasaï Central and 12 in Kasaï Oriental) and revised after an evaluation of the pilot test. The finalised pedagogical notes were used to update the FP content of the first-year midwifery teaching module. Both teachers and students of the first year of the midwifery course approved of the updated module, leading to a recommendation for additional training in subsequent years of the midwifery course.^10^

## What were the outcomes of the change in practice?

The PPFP counselling tool and MEC Wheel are integrated into the training programmes for midwives in three ISTMs (Kasaï, Kasaï Central, Kasaï Oriental) in the Kasaï region. To date, three batches of midwifery students have been trained using the revised curriculum. These trained midwives provide FP counselling and services on returning to their communities. SANRU, United Nations Population Fund (UNFPA) and other partners provided contraceptives in the three provinces.

To monitor the progress on PPFP uptake, three indicators were incorporated into District Health Information Software 2 (DHIS2): (i) the number of women who gave birth and were counselled on FP methods, (ii) the number of new acceptors of FP methods in the postpartum period and (iii) the number of new acceptors of FP methods post-abortion. table 2 presents a comparison of these three indicators and the percentage increase between January–June 2019 to 2023 in the three provinces.

**Table 2:** Comparison of postpartum family planning indicators and their percentage change from 2019 in the three provinces (January–June 2019 to 2023). Source: District Health Information Software 2 (DHIS2)

PPFP indicators from January to June	Kasaï					Kasaï central					Kasaï oriental				
	2019	2020	2021	2022	2023	2019	2020	2021	2022	2023	2019	2020	2021	2022	2023
Women who gave birth and were counselled on FP methods (n (%))	88 371	90 484 (2.4)	97 560 (10.4)	100 279 (13.5)	107 181 (21.3)	86 486	90 963 (5.2)	95 933 (10.9)	92 375 (6.8)	102 627 (18.7)	57 298	67 364 (17.6)	75 867 (32.4)	91 341 (59.4)	92 869 (62.1)
New acceptors of FP methods postpartum (n (%))	32 189	34 841 (8.2)	37 294 (15.9)	45 083 (40.1)	55 075 (71.1)	19 424	20 581 (6.0)	22 851 (17.6)	23 851 (22.8)	46 297 (138.0)	8294	19 892 (140.0)	18 238 (120.0)	21 814 (163.0)	27 023 (226.0)
New acceptors of FP methods post-abortion (n (%))	879	1180 (34.2)	1730 (96.8)	1662 (89.1)	2673 (204.0)	740	722 (−2.4)	928 (25.4)	828 (11.9)	1665 (125.0)	477	481 (0.8)	420 (−11.9)	537 (12.6)	702 (47.2)

FP, family planning; PPFP, postpartum family planning.

Over the course of 5 years (2019–2023), the number of new PPFP acceptors in Kasaï province increased by 71%, by 138% in Kasaï Central and 226% in Kasaï Oriental. Districts reported that better communication between midwives and clients through improved counselling may have contributed to increased PPFP uptake. Similarly, the number of new FP acceptors in the post-abortion period increased by 204% in Kasaï province, 125% in Kasaï Central and 47.2% in Kasaï Oriental. The number of women who were counselled on FP methods after birth increased in all three provinces (by 21% in Kasaï, 18.7% in Kasaï Central and 62% in Kasaï Oriental). There was also an increase in the uptake of PPFP among the women who received counselling, indicating an improvement in the quality of counselling. An example of this is in Kasaï province, where the uptake of PPFP among counselled women increased from 36% in 2019 to 51% in 2023. The project showed that incorporating FP training into the midwifery curriculum increases the uptake of PPFP and suggests scalability across different settings. Moreover, the updated curriculum was used to train 90 nurses in the Kasaï region to become midwives.

In Kasaï Central, the trained Reproductive Health Coordinator continues to scale up the intervention through supportive supervision of FP services in health facilities, focusing on nurse managers and maternity managers throughout the province.

## Lessons learned and key challenges

Integrating FP training into the undergraduate midwifery curriculum enabled midwives to practise FP competently immediately after completing their university education. Training midwifery teachers helped improve the learning experience of their students. The integration of the PPFP counselling tool in the ISTMs revealed the lack of basic knowledge of FP, particularly of PPFP, among teachers and midwives.

Key challenges include the need to regularly monitor the approach by all stakeholders, the need to ensure its sustainability, and the increase in the number of hours dedicated to FP classes.

## Conclusions

Incorporating training on FP counselling into the midwifery curriculum and training midwives on the use of a PPFP counselling tool and MEC Wheel can improve PPFP uptake. Scaling up this approach to include additional training for midwives, nurses and doctors can further improve the unmet need for FP in the DRC.

**Key message**

- In the Democratic Republic of Congo, postpartum family planning (PPFP) uptake increased following midwives' training based on a curriculum that included how to use a PPFP counselling tool and the Medical Eligibility Criteria for Contraceptive Use (MEC) Wheel.
